# Mobile Health for Obsessive-Compulsive Disorder: Patients' Preferences and Perception of Patient-Centeredness

**DOI:** 10.62641/aep.v53i1.1715

**Published:** 2025-01-05

**Authors:** Ana Isabel Araújo, Ana Telma Pereira, Isabel Catarina Duarte, Remy Cardoso, Miguel Castelo-Branco, António Macedo

**Affiliations:** ^1^Institute of Psychological Medicine, Faculty of Medicine, University of Coimbra, 3004-504 Coimbra, Portugal; ^2^Coimbra Institute for Biomedical Imaging and Translational Research (CIBIT), University of Coimbra, 3000-548 Coimbra, Portugal; ^3^Institute for Nuclear Sciences Applied to Health (ICNAS), University of Coimbra, 3000-548 Coimbra, Portugal; ^4^Department of Psychiatry, Coimbra Hospital and University Centre, 3004-561 Coimbra, Portugal; ^5^NOVA Medical School, Faculdade de Ciências Médicas, Universidade NOVA de Lisboa, 1169-056 Lisboa, Portugal

**Keywords:** obsessive-compulsive disorder, mHealth, patient preferences, patient-centered care

## Abstract

**Background:**

The increasingly fast development of mobile health technologies holds significant value for individuals dealing with mental health conditions. However, inadequate consideration of patients' preferences and expectations undermines real-world outcomes, including sustained adherence. Driven by the belief that specific characteristics, such as youth and higher education, of individuals with obsessive-compulsive disorder make them suitable for digital adoption, we investigated mHealth-related desirability factors within this patient group.

**Methods:**

Fifty-one conveniently selected adults with obsessive-compulsive disorder filled in a self-report questionnaire about symptom self-management preferences, with an emphasis on assessing mobile health options and perceptions of patient-centeredness.

**Results:**

The smartphone phone app emerged as the top choice of most of the sample for receiving information about symptom status (82.4%), obtaining general information about obsessive-compulsive disorder (74.5%), and symptom self-registration (66.7%), with no significant effect of sex or living location. Although only 23.5% of participants were using a health-related app, most expressed interest in using it for receiving symptom management tips (98.1%), medical advice (94.2%), symptom evolution updates (90.2%), lifestyle information (92.2%), medication tracking (88.2%) and short symptom self-reports (90.2%). Median expectations regarding mobile health's impact on patient-centeredness, satisfaction, and adherence were positive or very positive.

**Conclusions:**

Our data confirm that individuals with obsessive-compulsive disorder exhibit strong inclinations and optimistic expectations toward technology-based solutions. We highlight some of the preferences within this patient group, which can inform the design of practical, real-world applications.

## Introduction

From biomedical to patient-centered perspectives of medicine, it is becoming increasingly evident that “one size does not fit all”, and targeted interventions enhance clinical outcomes. This paradigm shift creates new hope for treating diseases previously considered resistant or untreatable. Notable examples come from areas such as oncology and cardiovascular medicine, where healthcare providers use analytical, histological, and radiological parameters in disease staging and patient profiling to determine the most appropriate treatment for each individual [1]. Personalized care approaches promise to revolutionize the way we practice medicine [1]. But how will the field of psychiatry keep pace with these advances, considering our dearth of tools capable of translating symptoms into objective measures [2]?

Smartphones and other connected devices now offer novel opportunities to capture objective data about individuals’ lived experiences [3, 4]. This approach, termed ecological momentary assessment, enables the collection of temporally dynamic and environmentally influenced features of cognition, emotion, and behaviour, all of which are central in psychiatric manifestations [4]. Among other options, mobile applications (apps), given their widespread availability, represent a particularly valuable tool for precision medicine [2, 5, 6]. Technological solutions may also foster patient-centered practices and empower individuals to manage their own health through digitally enabled care pathways [6]. The term mobile health (mHealth) emerged in this context to encompass the adoption of mobile devices to support clinical practice [7].

However, the collection of data and its integration into established clinical standards hinges on patient engagement, which is now suboptimal. A key challenge concerns our limited understanding of which persons are most suitable for using mHealth technologies and who will ultimately benefit from them [8, 9]. Although some evidence points to younger, higher-educated, male, and median-income individuals as preferential users [10], more efforts are necessary to delineate detailed profiles. The inclusion of patients’ preferences in technological development, another important factor for the effectiveness of these interventions [11], has also been very reduced [8]. For instance, Larsen *et al*. [12] evaluated the seventy-three most highly ranked mental health apps and found that only 14% reported user engagement in their development. Similarly, Cucciniello *et al*. [8] identified that only around one-third of the studies of mobile apps for chronic diseases reported user or healthcare professional involvement during the design stage, and few specified the process in detail. The disparity between the lack of reliable information on the desirability and user preferences of mHealth apps and their increasing availability on the market [7] is a major concern for health institutions and clinicians. High heterogeneity of sociodemographic characteristics across disorders may further influence and confound patterns of mHealth adherence, usage, and outcomes (Ventura *et al*., 2022, [13]). Thus, to achieve app specifications with the potential of translating into real-world advantages for patients, we advocate that studies need to focus on discrete patient segments.

Affecting 2.5–3% of the general population, obsessive-compulsive disorder (OCD) is a chronic condition that typically arises during childhood, adolescence, or early adulthood and causes considerable disability [14]. These life stages, while being challenging in terms of rapid change and adaptation, coincide with phases when individuals are more predisposed to using digital technologies [10]. The hallmarks of OCD include recurrent, intrusive, distressing, thoughts or images (obsessions) accompanied by time-consuming mental or behavioural rituals (compulsions). In addition to being costly, access to both initial and advanced treatments remains limited to central hospitals, resulting in geographical disparities. Furthermore, the current OCD treatment model follows a stepped-care approach, leading to a delay of approximately two years between seeking help and receiving adequate treatment [15]. To improve treatment efficiency, stratified care frameworks—tailoring treatment to individual patients—require objective data, such as that collected through mobile apps [14, 16].

All these considerations suggest that individuals with OCD, despite facing challenges similar to other patients, due to their characteristics, are uniquely positioned to benefit from mHealth solutions. In this line, our study investigates mHealth-related desirability factors, user preferences, and perceptions of patient-centeredness among individuals with OCD.

## Materials and Methods

### Participants

The reported analysis included 51 conveniently selected adults under clinical care at the OCD Treatment Unit of the Coimbra Hospital and University Centre. The diagnoses of OCD were performed by experienced psychiatrists and psychologists as the standard protocol from this Unit, according to the fifth edition of the Diagnostic and Statistical Manual of Mental Disorders [17]. Participants were not included if, based on self-reported responses, they had an active comorbidity namely depression, anxiety, alcohol dependency, or behavioral addictions, or if OCD was not the primary diagnosis. This study was conducted under the Declaration of Helsinki. We obtained ethical approval from the Coimbra Hospital and University Centre Ethics Committee (OBS.SF.151/2023) and all participants filled out an online informed consent after an explanation of the study procedure and aims.

### Procedure

Participants were contacted through a phone call or approached while waiting for their medical/psychology appointment at the hospital. The investigator explained that the team was developing a smartphone app for OCD symptom monitoring and psychoeducational material delivery. Other planned functionalities such as psychotherapeutic tips to manage OCD symptoms were also mentioned. The investigator clarified to the participants that, to define the following steps in the app development, it was important for the research team to know whether they expected a positive, neutral, or negative impact in their lives from using an app like that. Individuals who provided consent were subsequently administered a self-reported questionnaire, delivered via e-mail.

### Self-Reported Protocol

The protocol included questions about socio-demographic characteristics, the clinical history, and the following self-reported questionnaires:

#### Flashcard Questions about OCD Self-Management Preferences

These questions evaluated symptom management preferences in three domains: symptom self-registration, receiving individualized information about symptom evolution, and receiving general information about OCD. The possible options (memory, notebook, book, TV, website) for each question were illustrated using flashcards. Although no psychometric assessment has been conducted, flashcard quizzes constitute a common method for product-costumer fit evaluation [18].

#### mHealth Questionnaire for OCD

This questionnaire included 10 mHealth-related questions, namely (a) two questions about general mobile phone use, (b) seven questions about patients’ preferences of symptom monitoring and management through a mobile app, filled on a 5-point Likert scale (from 1, strongly disagree to 5 strongly agree) and (c) one question about the willingness to answer a short app-delivered self-report questionnaire (2–3 minutes) about their symptoms. The questions were adapted from Schuuring *et al*. (2016) [19] to align with the specific context of individuals with OCD. Neither the original nor the Portuguese versions were validated.

#### Patient Perception of Patient-Centeredness (PPPC-16) Questionnaire

The original Patient perception of patient-centeredness (PPPC) [20] questionnaire is composed of 14 items to measure the patient’s perception of patient-centered care regarding the last medical appointment, using a 4-point Likert scale (from 1, not at all to 4, completely). In the present study, we used the Portuguese version of the PPPC (PPPC-16) [21], which included two additional items (15 and 16) based on Mead and Bower’s [22] biopsychosocial perspective of patient-centeredness. The Portuguese version of the PPPC (i.e., the PPPC-16), showed good psychometric properties (reliability and validity) for both two factors (Empathy and Patient involvement) and one-factor structure [21]. The PPPC-16 is usually supplemented by three additional questions about the patient’s satisfaction with the consultation and adherence to pharmacological and non-pharmacologic treatments [23]. These questions were included in the questionnaire, but they were not considered for the global and subscales scores of the PPPC-16.

#### Impact of a mHealth App for OCD in Patient Perception of Patient-Centeredness 

This questionnaire was adapted from the PPPC-16 [20, 21, 24]. Participants were asked to fill in the 16 questions of the PPPC-16 plus three additional items about patient satisfaction and adherence, considering the expected impact of a mHealth app for OCD management, using a 5-point Likert scale (from 1, very negative impact to 5, very positive impact). At the beginning of the questionnaire, we included a tip instructing participants to remember their last consultation and imagine how it would have been if they were using this type of app.

#### Final Open Question (Optional)

We asked participants to use their own words to describe their expectations about using a mobile app to manage OCD symptoms.

All the adaptations of the original questionnaires were made by our team including a PhD psychologist with more than two decades of experience in psychometric evaluation (ATP) and two psychiatrists (AM and AIA).

### Statistical Analysis

For the statistical analysis, we used the Statistical Package for Social Sciences, version 26 (SPSS®, Chicago, IL, USA). We first performed descriptive statistics to characterize the symptom management preferences of our sample and the expected impact of using a mHealth app. To analyze the association between participants’ characteristics (age, sex, level of education, and disease duration) and mHealth preferences, we dichotomized this variable into “smartphone” and “others” (book, website, television, memory, notebook) and applied chi-square and Mann-Whitney’s tests according to the type of variable in the analysis.

## Results

### Participants Sociodemographic and Clinical Characteristics 

Fifty-one adults (62.7% women) with OCD participated. A single individual diagnosed with OCD was initially contacted but then excluded from the study due to non-engagement with email correspondence. The median age was 32 (range 19–59), 51% had concluded higher education and 39.2% had secondary education. Living location was classified as urban by 49.2% of the sample. Most of the participants (84.3%) denied having comorbid psychiatric diagnoses. The median age of OCD onset was 18 (range 4–40) years and OCD diagnoses were received 3 (median; range 0–26) years later. The median OCD duration was 15 (range 1–38) years. Most of the patients were being treated with cognitive-behavioural therapy, antidepressant medication, and, less frequently, antipsychotics (Table 1).

**Table 1.** **Participants’ characteristics (*n* = 51)**.

		Individuals with OCD
		(*n* = 51)
Sex (% women)		62.7% (*n* = 32)
Age (mean; range)		32; 19–59 years
Educational level		
	Higher education or higher	50.98% (*n* = 26)
	Secondary education or lower	49.02% (*n* = 25)
Living location		
	Urban	49.02% (*n* = 25)
	Rural	50.98% (*n* = 26)
Comorbid psychiatric diagnoses (yes)		15.7% (*n* = 8)
Age at OCD onset (mean; range)		18.4; 4–40 years
OCD duration (mean; range)		15.09; 1–38 years
OCD medication (yes)		92.16% (*n* = 47)

The presented data was based on the participants’ self-report. OCD, obsessive-compulsive disorder.

### Symptom-Management Preferences

**Fig. 1.**
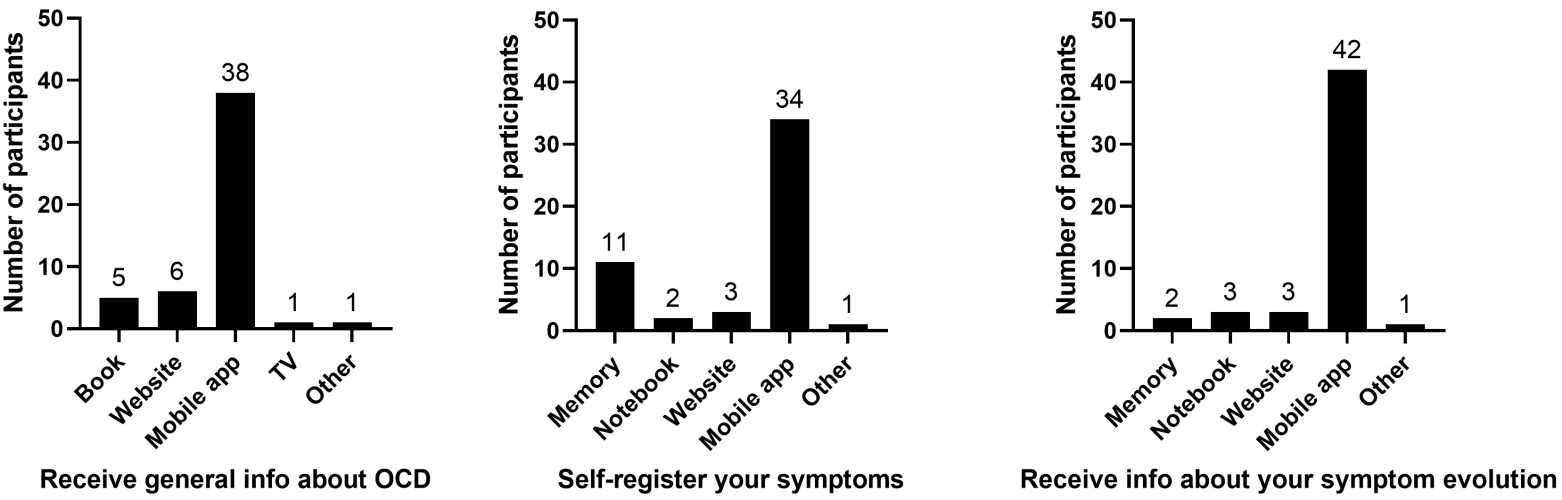
**Symptom-management preferences of adults with obsessive-compulsive disorder (OCD) (*n* = 51)**. The numbers above the bars represent the number of participants.

**Fig. 2.**
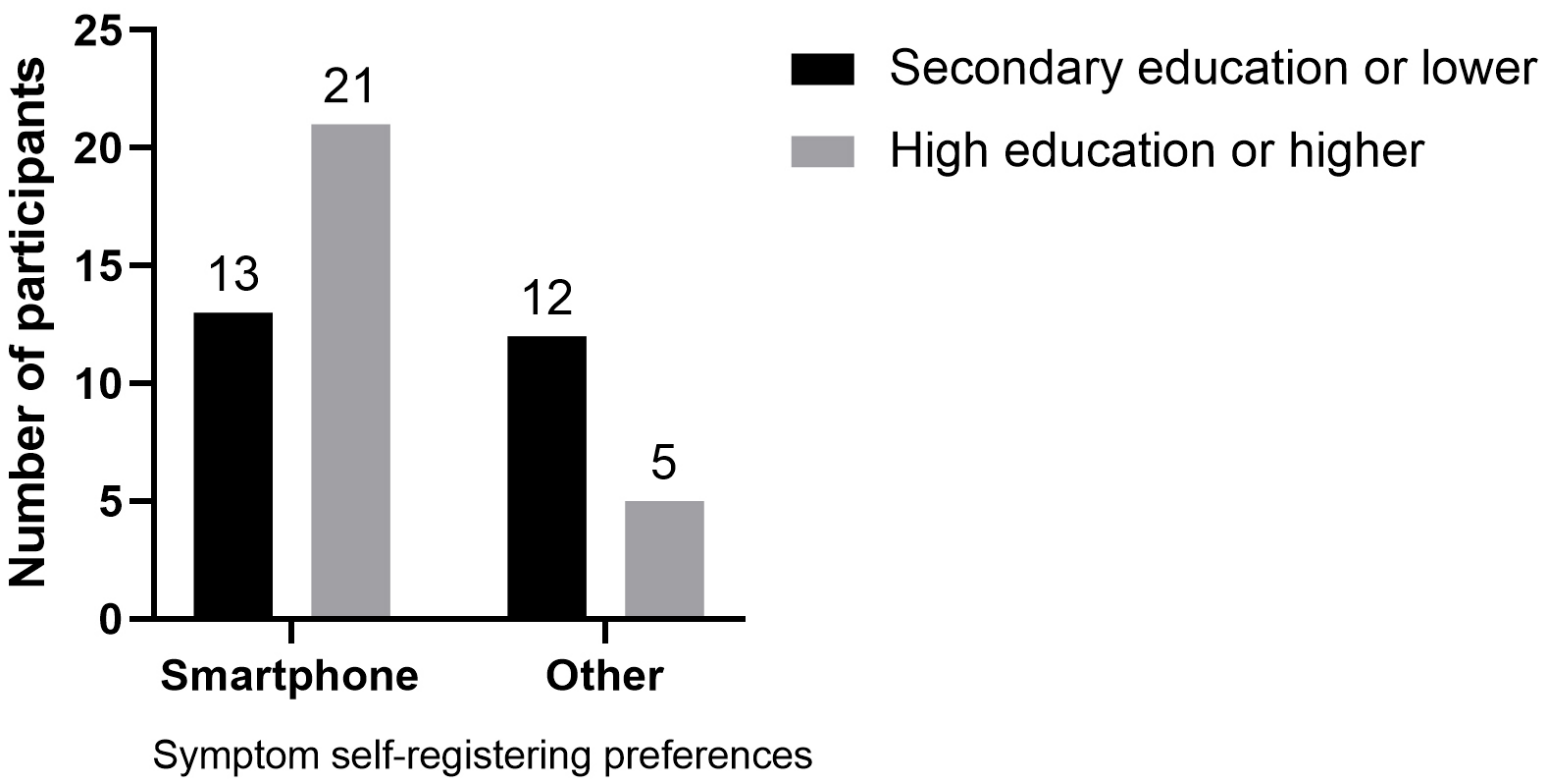
**Symptom self-registering preferences (smartphone *versus* other [memory, notebook, website, other]) according to the level of education (secondary education or lower *versus* high education or higher; *n* = 51)**. There was a significant association between having a higher education level and choosing “smartphone” as the preferred means for symptom self-registration (*Pearson’s chi-square* = 4.747, *p* = 0.029; *Cramer’s V *= 0.305, *p* = 0.029). The numbers above the bars represent the number of participants.

Within the available options (memory, notebook, book, TV, website, mobile app), most of the participants reported a preference for using a mobile app to receive general information about OCD (74.5%), self-register their symptoms (66.7%) and receive information about their symptom status (82.4%; Fig. 1). Compared with the other options to record OCD symptoms, participants who preferred using a mHealth app also had higher education levels (*Pearson’s chi-square* = 4.747, *p* = 0.029; *Cramer’s V* = 0.305, *p* = 0.029, uncorrected; Fig. 2). There was no association between the other symptom-management preferences and education level. Sex, living location, and disease duration also did not affect symptom management preferences. For detailed information, please see **Supplementary Tables 1,2,3,4**.

### Desirability and Expected Impact of a mHealth Mobile App

**Fig. 3.**
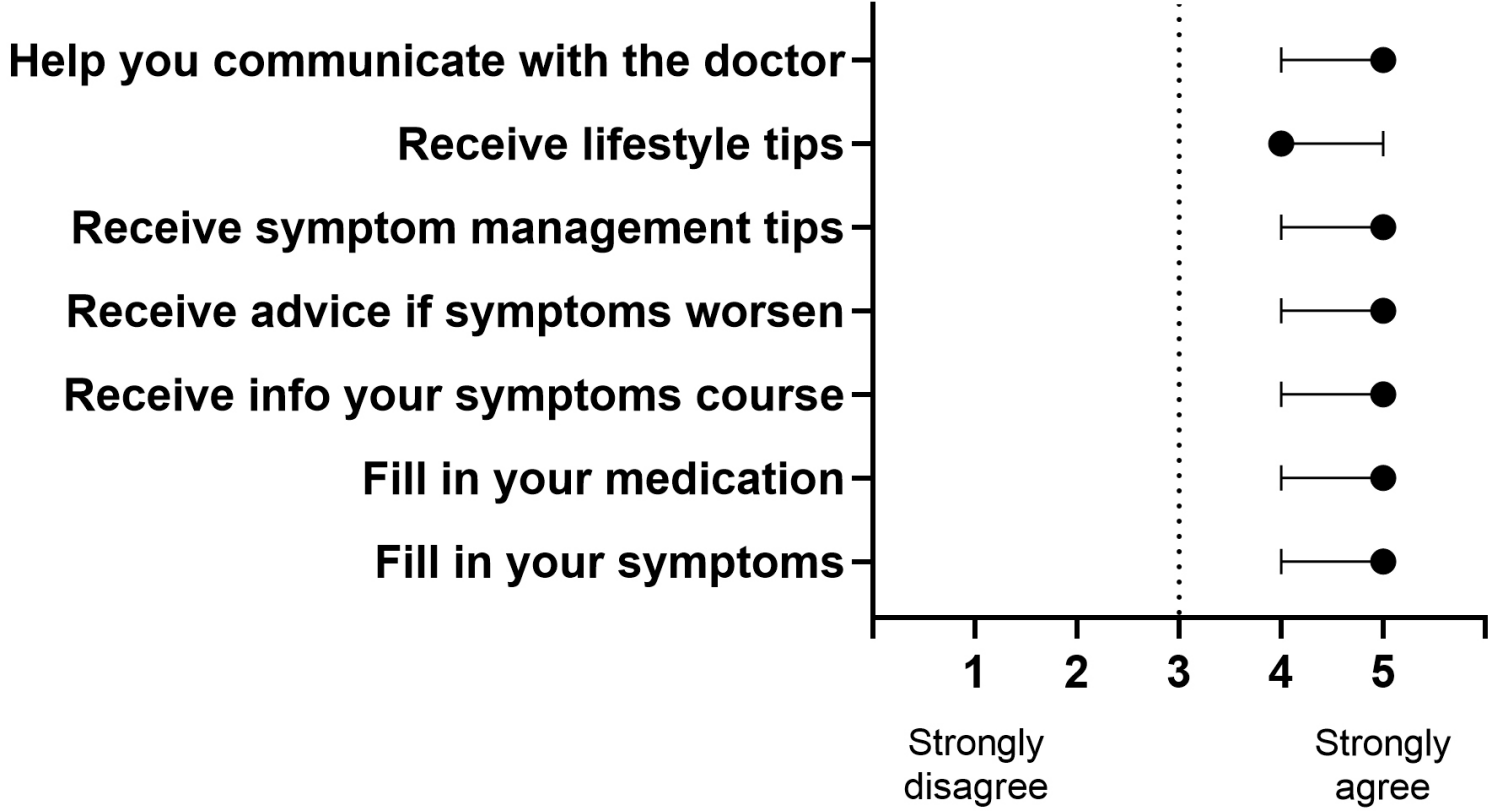
**Desirability of a mHealth app for individuals with obsessive-compulsive disorder**. The black dot represents the median scores, and the error bars represent the interquartile range for each item. The scale, from 1 (strongly disagree) to 5 (strongly agree), refers to how much the participants would like to use each mobile app functionality.

All participants owned a smartphone but only 23.5% were using a health-related app. The majority reported that they would like (agree and strongly agree) to use a mHealth app to receive tips about symptom management (98.1%), medical advice in the case of clinical worsening (94.2%), and information about symptom evolution (90.2%) and lifestyle (92.2%) (Fig. 3). Most of the sample was also willing (agree and strongly agree) to fill in the medication status (88.2%) and a short self-report questionnaire about their symptom status (90.2%). Of that 90.2%, the majority (55.3%) were disposed to answer the questionnaire one time per week, 14.9% on alternating days, 17% daily, and 12.8% twice a day. Most of the participants (90.2%) agreed or strongly agreed that using mHealth would facilitate patient-doctor communication. Excepting lifestyle information (median 4), all the other functionalities scored the highest value (median 5), from 1 (strongly disagree) to 5 (strongly agree; Fig. 3), regarding whether participants would like to use those mobile app functionalities. Twelve participants responded to the open question. In ten, out of twelve answers, participants stated an expected positive impact of using a mobile app for OCD. The other two answers were considered neutral regarding the main point. There, the participants expressed concerns about the need to ensure privacy and prevent stigma when using this type of tool. Four of the responses focused on psychoeducational and treatment aspects such as real-time symptom management and consolidation of the psychotherapy principles and practices between consultations. Three responses emphasized the possibility of enhancing access to healthcare providers (namely via online consultations) and medical prescriptions as well as medication reminders. There were also three responses about the potentially positive impact of real-time symptom monitoring on symptom prevention and management and communication with the physician.

### Perception of Patient-Centeredness

The median total score in the PPPC-16 was high (45 out of 64; range 28–60), as well as in the subscales “Empathy” (30 out of 44; range 20–40) and “Patient-involvement” (15 out of 20; range 6–20). The median of all the PPCD-16 items was 3 (out of 4), indicating that overall, participants mostly believed that the clinician had been patient-centered during the previous appointment in interaction-specific components, such as exploring disease and illness, understanding the whole person, and finding common ground. Considering the additional questions about the patient’s satisfaction with the consultation and adherence to treatments, patients were very satisfied (median 5 out of 6), and declared adhering always and most of the time to the prescribed medication (median 6 out of 6), and other medical recommendations (median = 5 out of 6), respectively.

### Expected Impact of a mHealth Mobile App on Perception of Patient-Centeredness

The median expected impact of using an app for OCD was positive (median 4 out of 5 on 11 items) or very positive (median 5 out of 5 on 5 items) on all the items of the PPPC-16, indicating that, globally, participants expected an improvement in their perception that the medical encounter was patient-centered. The median impact on the PPPC-16 was 64 out of 80 (range 45–75) for the total score, 43 out of 55 (range 30–50) for the “Empathy” subscale, and 21 out of 25 (range 15–25) for the “Patient-involvement” subscale. The expected impact was positive (median 4 out of 5) for patient satisfaction and adherence to medical recommendations and very positive (median 5 out of 5) for medication adherence.

## Discussion

The integration of digital apps into healthcare holds significant promise for fostering evidence-based and patient-centered practices [6, 7, 25], particularly in the field of psychiatry [5]. Governmental institutions are now developing norms and legislation to support and regulate the adoption of mHealth technologies [25]. A positive impact on health systems’ accessibility and economic viability is also expected [25, 26, 27].

In the context of mental health, mobile apps provide a unique opportunity to capture objective information about symptoms, facilitating bidirectional interactions between doctors and patients. However, validation of the collected measures for incorporation into medical decision algorithms necessitates that patients actively use these tools. Such engagement depends on users perceiving tangible improvements in their lives from these interventions. Our study focused on individuals with OCD, as this patient group is uniquely positioned to benefit from digital innovations. So, we asked patients about their preferences and expectations regarding a hypothetical mobile app for symptom monitoring and management.

App-delivered symptom monitoring and management functionalities, such as educational tips and medical advice, were highly valued. This suggests that patients with OCD hold optimistic expectations concerning mHealth and highlights the central role of clinicians in this process. Compared with the other modalities (book, TV, website, other), most participants preferred using a mobile app as the first choice to receive general information about OCD. Getting information from books or websites was the top choice for a minority of the sample. Thus, customization of the existing clinical instruments (e.g., educational resources, intervention programs, and self-reported questionnaires) for smartphone adaptation should be a priority for clinicians and developers, as the currently available options are very limited [12, 28].

Regarding smartphone-delivered self-report questionnaires for OCD, these tools have already demonstrated good sensitivity and adequate specificity for detecting symptoms, when compared with a structured clinical interview [28]. In the present study, most of the participants preferred using a mobile app (*versus* memory, notebook, or website) for symptom self-registering and were willing to fill out a short symptom questionnaire at least weakly. Notably, an even greater proportion expressed a preference for receiving information about their symptom status through the smartphone. This suggests that whereas most patients with OCD favored the smartphone to both register and obtain monitoring feedback, there was a minority that would like to keep track of their symptoms through this platform without having to input any clinical data. Individuals with higher education degrees were those who mostly chose the smartphone as the first option for symptom self-registering, which accords with previous research [10].

Our findings point to passive data collection as an important feature to include in mHealth solutions for OCD, at least for specific patient segments, such as those with lower academic levels. This modality of health assessment has minimal interference with the user’s daily living and can provide continuous quantitative data on behaviours that are relevant to psychiatric conditions [29, 30, 31]. Emerging evidence suggests that digital tools are capable of capturing many OCD-related features. For instance, WashSpot—a neural-network based method—can assess compulsive handwashing using inertial motion sensor data from smartwatches [32]. Obsessive slowness, an increase in the time spent in the same location due to OCD, has been digitally evaluated with the GPS [33]. Other potential applications include the assessment of ritualistic behaviours through geolocation and accelerometer systems, and social avoidance using connectivity with other devices. Conversely, to be useful in medical practices, sensor-based parameters and supporting technologies require further validation and testing [31]. For instance, when applying smartphones and wearable devices as medical devices, differences between brands and models become a potential concern. Thus, researchers and the industry need to develop and implement standards across different technological systems. It is also important for future studies to elucidate the exact relationship between clinical symptoms and sensor data, in both laboratory and naturalistic settings. This is not without nuances. In the case of OCD, avoidance behaviours may confound the interpretation of sensor data, because they lead to artificial decreases in the severity of compulsive rituals. Thus, to generate meaningful information about psychiatric symptoms, low-level features derived from sensor data will have to be aggregated into high-level behavioural markers [31].

Although only one-quarter of the participants in the present study had health-related apps installed on their smartphones, the majority expressed a propensity to use mHealth as their primary choice for symptom status registration and feedback. This preference was independent of the patient’s living location. Individuals residing in rural areas already face geographical disparities in proximity to central hospitals [10] and are at risk of further disadvantages in digital transition due to limited access to technological infrastructure in those regions [10, 25]. We thus suggest that, although rural-dwelling patients with OCD may not have equal opportunities, they exhibit similar mHealth preferences as those living in urban areas. The participants’ sex, age, and disease duration did not influence their preferences, reinforcing that mHealth is desirable across a wide spectrum of OCD patient profiles.

The present findings indicate that while OCD’s characteristics present a good fit for mHealth solutions, patients are not currently utilizing these tools. Considering reports of inadequate digital literacy across many developed countries [25], we hypothesize that low digital adoption in our sample may be attributed, at least partially, to individuals with OCD lacking the necessary digital competencies. In response to this reality, international entities are now advocating for investment in digital literacy and capacity building [25], from which OCD patients may also benefit. Given the influential role that healthcare providers play in patient health behaviour, their recommendations are vital for patients to trust and adhere to medical apps [6]. However, there are still many factors hindering clinicians from prescribing mHealth technologies as part of the usual clinical practice. These include the absence of globally approved standards for app prescription and integration into existing workflows (e.g., pharmacological prescription systems), as well as the need for digital health training [10]. A recent report identified an effect of the user’s perceived product value on actual usage of mHealth apps [34], underscoring the utility of understanding patient’s preferences.

In addition to providing a means of assessment, smartphones can also deliver individually tailored actions [2], enabling several opportunities to make healthcare more patient-centered [35]. Patient-centered care is a philosophy that encourages the focus on the patient as a whole person with individual preferences and prioritizes effective clinical communication as a cornerstone for shared decision-making [20]. Participants in our study reported good perceptions of patient-centeredness and high satisfaction with their current clinical follow-up. Considering this baseline, they anticipated a positive impact from mHealth on the perception of patient-centeredness, satisfaction, and adherence to medical recommendations. Specifically, using a mobile app for OCD symptom management was expected to enhance patient-physician communication, promote active engagement in therapeutic discussions, and foster shared decision-making [20]. All these factors promote patient satisfaction, improved self-management, and treatment adherence, and lead to lower healthcare costs [20].

The enthusiasm surrounding the adoption and dissemination of technology-driven medical care must be balanced by addressing concerns related to clinical safety, data protection, security, usability, and accessibility [6, 25]. The participants in our study identified privacy and security as major factors to be addressed before using mHealth apps. This underscores the importance of medical device validation processes and data management transparency. In addition, it aligns with the typical OCD profile marked by threat overestimation and harm avoidance behaviours [36]. Therefore, beyond ensuring technical reliability and addressing data privacy concerns, developers should consider incorporating safety-enhancing features into the user experience strategies of OCD app design.

Other user-experience factors warranting investigation include gamification and community-building elements, as they may promote engagement and sustained adherence, foster feelings of patient-centeredness, and ultimately facilitate behavioral change. The inclusion of these components in mHealth development has been infrequent [8], and presents a challenge for developers. In OCD, reinforcement learning impairments [37] may complicate the adequate implementation of gaming schemes based on negative and positive valence stimuli dichotomies and rewards. If not applied judiciously, gamification may risk exacerbating OCD’s compulsive behaviors and anxiety. Difficulties in impulse control [38, 39] are relatively frequent in OCD and may also increase the complexity of health-related app design strategies. It is thus crucial to understand the specific mHealth usage patterns of OCD patients and apply this knowledge to improve app development. The initial tests in real-world patients represent unique opportunities to identify limitations and introduce necessary modifications.

A limitation of our study was its exclusive focus on individuals receiving regular in-person clinical follow-ups, as patients with limited access to expert clinical advice may derive the greatest benefits from using mHealth [40]. Thus, we recommend that future studies explore mHealth preferences in larger samples, encompassing a wider range of socioeconomic statuses and age groups.

## Conclusions

We addressed a literature gap by identifying individuals with OCD as suitable candidates for using mHealth tools. By considering user perspectives—an area that has been understudied—our findings, regarding a hypothetical app for OCD, indicate high desirability in terms of expected impact on symptom management, patient-centeredness, and overall satisfaction. We demonstrated that individuals with OCD, regardless of their demographic and educational characteristics, exhibit strong inclinations and optimistic expectations toward the adoption of technology-based solutions. So, our study provides relevant insights for integration into mHealth app development specifically for OCD patients. Furthermore, we emphasize that the potential of digital interventions to translate into real-world functional improvements relies on collaborative efforts among developers, researchers, clinicians, and patients.

## Availability of Data and Materials

The datasets generated and/or analyzed during the current study are not publicly available but are available from the corresponding author upon reasonable request.

## Supplementary Material

Supplementary material associated with this article can be found, in the online version, at https://doi.org/10.62641/aep.v53i1.1715.
