# Supplementary material for: Mobile Health for Obsessive-Compulsive Disorder: Patients' Preferences and Perception of Patient-Centeredness
Source: Actas Esp Psiquiatr. 2025 Jan 5;53(1):100–9. doi: 10.62641/aep.v53i1.1715 (PMC11726213; doi:10.62641/aep.v53i1.1715)
Supplement: Supplementary file 1 [file ActEsp-53-1-100-109-s1.docx]

Mobile health for obsessive-compulsive disorder: users’ preference and perception of patient-centeredness

*Supplementary material*

**Supplementary Table 1. Associations between sex and symptom management preferences of individuals with obsessive-compulsive disorder (*n* = 51)**

|  | | Sex | |  |  |
| --- | --- | --- | --- | --- | --- |
| Symptom management preferences | | Women  (*n* = 32) | Men  (*n* = 19) | Pearson’s chi-square | *p*-value |
| Receive general info about OCD | Smartphone  (*n* = 38) | 22 | 16 | 1.5 | 0.221 |
|  | Other  (*n* = 13) | 10 | 3 |  |  |
| Self-register your symptoms | Smartphone  (*n* = 34) | 21 | 13 | 0.042 | 0.838 |
|  | Other  (*n* = 17) | 11 | 6 |  |  |
| Receive info about your symptom evolution | Smartphone  (*n* = 42) | 25 | 17 | 1.057 | 0.304 |
|  | Other  (*n* = 9) | 7 | 2 |  |  |

**Supplementary Table 2. Associations between living location and symptom management preferences of individuals with obsessive-compulsive disorder (*n* = 51)**

|  | | Living location | |  |  |
| --- | --- | --- | --- | --- | --- |
| Symptom management preferences | | Rural  (*n* = 27) | Urban  (*n* = 24) | Pearson’s chi-square | *p*-value |
| Receive general info about OCD | Smartphone  (*n* = 38) | 20 | 18 | 0.006 | 0.940 |
|  | Other  (*n* = 13) | 7 | 6 |  |  |
| Self-register your symptoms | Smartphone  (*n* = 34) | 17 | 17 | 0.354 | 0.552 |
|  | Other  (*n* = 17) | 10 | 7 |  |  |
| Receive info about your symptom evolution | Smartphone  (*n* = 42) | 20 | 22 | 2.706 | 0.1 |
|  | Other  (*n* = 9) | 7 | 2 |  |  |

**Supplementary Table 3. Associations between education level and symptom management preferences of individuals with obsessive-compulsive disorder (*n* = 51)**

|  | | Education level | |  |  |
| --- | --- | --- | --- | --- | --- |
| Symptom management preferences | | Secondary education or lower  (*n* = 32) | High education or higher  (*n* = 19) | Pearson’s chi-square | *p*-value |
| Receive general info about OCD | Smartphone  (*n* = 38) | 18 | 20 | 0.163 | 0.687 |
|  | Other  (*n* = 13) | 7 | 6 |  |  |
| Self-register your symptoms | Smartphone  (*n* = 34) | 13 | 21 | 4.747 | 0.029 |
|  | Other  (*n* = 17) | 12 | 5 |  |  |
| Receive info about your symptom evolution | Smartphone  (*n* = 42) | 20 | 22 | 0.187 | 0.666 |
|  | Other  (*n* = 9) | 5 | 4 |  |  |

**Supplementary Table 4. Associations between disease duration and symptom management preferences of individuals with obsessive-compulsive disorder (*n* = 47)**

| Symptom management preferences | | Disease duration  mean rank | *Z* (Mann-Whitney) | *p*-value |
| --- | --- | --- | --- | --- |
| Receive general info about OCD | Smartphone  (*n* = 35) | 25.1 | -0.941 | 0.347 |
|  | Other  (*n* = 12) | 20.79 |  |  |
| Self-register your symptoms | Smartphone  (*n* = 32) | 22.02 | -1.451 | 0.147 |
|  | Other  (*n* = 15) | 28.23 |  |  |
| Receive info about your symptom evolution | Smartphone  (*n* = 38) | 22.43 | -1.611 | 0.107 |
|  | Other  (*n* = 9) | 30.61 |  |  |
